# Highly localized interactions between sensory neurons and sprouting sympathetic fibers observed in a transgenic tyrosine hydroxylase reporter mouse

**DOI:** 10.1186/1744-8069-7-53

**Published:** 2011-07-27

**Authors:** Wenrui Xie, Judith A Strong, Juxian Mao, Jun-Ming Zhang

**Affiliations:** 1 Pain Research Center, Department of Anesthesiology, University of Cincinnati College of Medicine, Cincinnati, OH 45267-0531, USA

**Keywords:** sympathetic sprouting, ectopic activity, spontaneous activity, spinal nerve ligation

## Abstract

**Background:**

Sprouting of sympathetic fibers into sensory ganglia occurs in many preclinical pain models, providing a possible anatomical substrate for sympathetically enhanced pain. However, the functional consequences of this sprouting have been controversial. We used a transgenic mouse in which sympathetic fibers expressed green fluorescent protein, observable in live tissue. Medium and large diameter lumbar sensory neurons with and without nearby sympathetic fibers were recorded in whole ganglion preparations using microelectrodes.

**Results:**

After spinal nerve ligation, sympathetic sprouting was extensive by 3 days. Abnormal spontaneous activity increased to 15% and rheobase was reduced. Spontaneously active cells had Aαβ conduction velocities but were clustered near the medium/large cell boundary. Neurons with sympathetic basket formations had a dramatically higher incidence of spontaneous activity (71%) and had lower rheobase than cells with no sympathetic fibers nearby. Cells with lower density nearby fibers had intermediate phenotypes. Immunohistochemistry of sectioned ganglia showed that cells surrounded by sympathetic fibers were enriched in nociceptive markers TrkA, substance P, or CGRP. Spontaneous activity began before sympathetic sprouting was observed, but blocking sympathetic sprouting on day 3 by cutting the dorsal ramus in addition to the ventral ramus of the spinal nerve greatly reduced abnormal spontaneous activity.

**Conclusions:**

The data suggest that early sympathetic sprouting into the sensory ganglia may have highly localized, excitatory effects. Quantitatively, neurons with sympathetic basket formations may account for more than half of the observed spontaneous activity, despite being relatively rare. Spontaneous activity in sensory neurons and sympathetic sprouting may be mutually re-enforcing.

## Background

Sensory neurons in the dorsal root ganglia (DRG) are normally not directly innervated by the sympathetic nerves. Sympathetic fibers in normal DRG are associated only with blood vessels. Sprouting of sympathetic fibers into the DRG has been described in several preclinical pain models [1-5]. Anatomically, this may include formation of dramatic "basket" structures in which sympathetic fibers form a dense plexus around individual somas (particularly of large diameter cells), as well as an increase in overall sympathetic fiber density in the cellular regions of the DRG. Basket structures have also been observed in DRGs from human neuropathic pain patients [6]. Sprouting occurs around both intact and axotomized cells [7], and can occur rapidly even in the absence of overt axotomy, for example in locally inflamed DRG [8], and mechanically compressed DRG [9].

The discovery of sympathetic sprouting in the DRG provided a possible anatomical basis for clinical syndromes of sympathetically maintained pain. Clinically, many chronic pain conditions such as complex regional pain syndrome are maintained or exacerbated by sympathetic activity in some patients, especially at earlier stages, and respond to various anti-sympathetic manipulations [10,11]. However, studies in this field using animal models have yielded sometimes contradictory results on the behavioral importance of the sympathetic-sensory connections. Many behavioral studies have shown that various forms of sympathectomy reduce or eliminate mechanical or thermal pain behaviors in rodents, but other studies been reached the opposite conclusion (see Table 1 in ref. [5]).

**Table 1:** Distribution of nociceptive markers in cells with and without sympathetic contacts

		Cells without Marker			Cells Expressing Marker				
				
Marker	Condition	Cells with Symp Fibers	Cells with Symp Baskets	Cells w/o Symp Contact	Cells with Symp Fibers	Cells with Symp Baskets	Cells w/o Symp Contact	Overall Percent Expressing Marker	Percent with Symp Basket or Fibers
**SP**	SNL	118	55	1576	168	114	558	32.4%*	17.6%*
	Normal	1	2	2637	2	0	622	19.1%	0.2%
**TrKA**	SNL	171	83	1267	109	68	237	21.4%*	22.3%*
	Normal	1	0	1431	3	1	1202	45.7%	0.2%
**CGRP**	SNL	134	50	1241	113	72	374	28.2%*	18.6%*
	Normal	3	0	1892	7	12	1429	43.3%	0.7%

Fixed DRG sections stained for TH and the indicated marker were prepared from normal DRG or on POD 3 after spinal nerve ligation. Number of cells in each category is indicated. N = 3 animals per group minimum. *, significant difference between normal and SNL (p < 0.0001; Chi Square test)

Few functional studies of abnormal sympathetic-sensory neuron connections have been reported. In general, these studies used extracellular fiber recording methods to measure increased spontaneous activity after addition of alpha adrenergic agonists or preganglionic stimulation in vivo [1,12,13]. These studies were not designed to detect absolute incidence of spontaneous activity, or to examine other measures of excitability such as rheobase and threshold, which require intracellular recording methods.

Recently, we demonstrated that sympathetic sprouts into the rat DRG at 3 days after spinal nerve ligation (SNL) originated primarily from the dorsal ramus of the spinal nerve [14]. This branch of the spinal nerve diverges from the ventral ramus close to the intervertebral foramen, and innervates posterior structures. The dorsal ramus is left intact in the spinal nerve ligation model, which involves ligation and (in some cases) transection of the much larger ventral ramus of the spinal nerve, in which fibers that innervate the paw and hindlimb travel. This finding allowed electrical stimulation of the dorsal ramus in the excised rat whole DRG preparation, in which microelectrode recordings of sensory neurons with attached axon segments and intact glial cell layers could be made. We showed that repeated stimulation of the dorsal ramus had excitatory effects on large and medium diameter sensory neurons, including increased spontaneous activity and decreased rheobase, beyond the excitatory effects induced by spinal nerve ligation per se. Experiments using sympathetic blockers, or in DRG in which sprouting was prevented, provided evidence that these excitatory effects were due to stimulation of sympathetic sprouts.

In the present study, we continue the development of techniques for functional study of abnormal sympathetic-sensory connections ex vivo. We used a transgenic mouse in which enhanced green fluorescent protein (EGFP) is expressed under control of the tyrosine hydroxylase (TH) promoter, the commonly used marker for sympathetic neurons. This allowed visualization of sprouted sympathetic fibers in the excised mouse DRG preparation. Microelectrode recordings were made in sensory neurons, based on an adaptation of the technique used in rat DRG. Sensory neurons were identified during the recording as being surrounded by extensive sympathetic basket or ring formations, having nearby but less elaborate sympathetic fibers, or having no nearby fibers. We report that excitability parameters differ markedly between these three classes of cells, suggesting that the sprouted sympathetic fibers have localized, functional excitatory interactions with nearby sensory neurons.

## Results

### Sympathetic sprouting onto the DRG occurred rapidly after SNL

**Figure 1:**
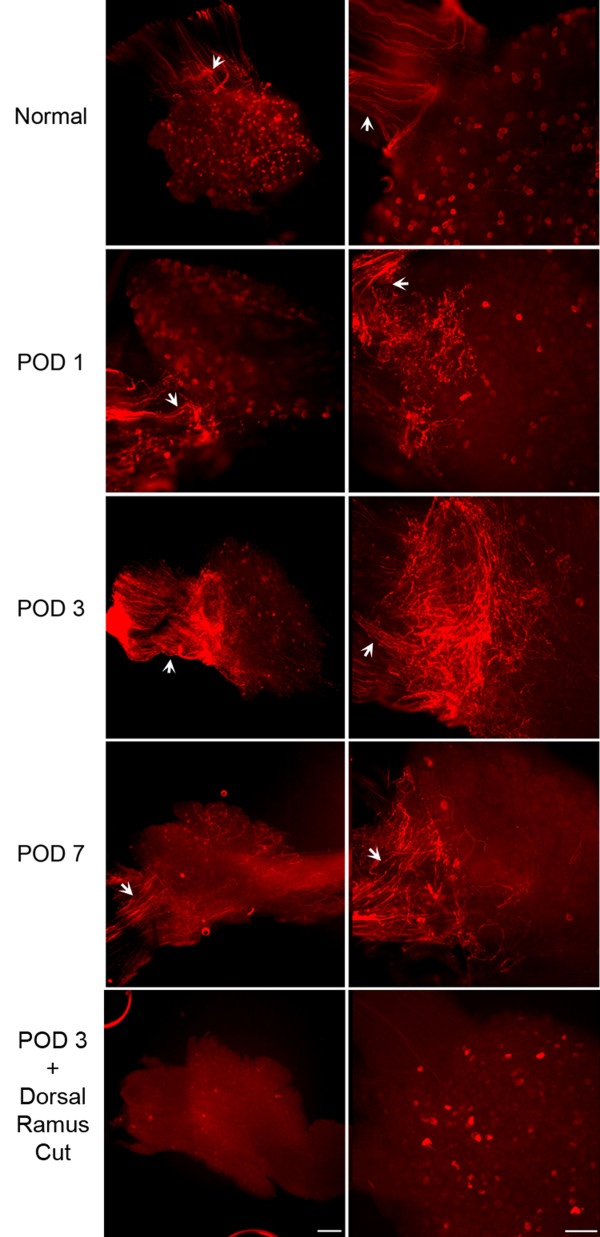
**Sympathetic sprouting onto DRG observed in fixed whole mount preparation after staining for TH**. TH staining is indicated in red using conventional immunohistochemistry. Arrows indicate the location of the dorsal ramus. In these low power whole mount views, the ventral and dorsal rami of the spinal nerve are to the left (in vivo the dorsal ramus would be perpendicular to the plane of the picture; it is flattened against the ventral ramus in the process of fixing the whole mount DRG preparation). Lower magnification (left; Scale bar = 100 μm) and higher magnification (right; scale bar 20 μm) views on the indicated POD are shown. "POD 3 + dorsal ramus cut", in this experiment the dorsal ramus was cut along with the ventral ramus; in all other POD figures the conventional spinal nerve ligation was done in which only the ventral ramus was cut.

Sprouting of sympathetic fibers in the transgenic pTH-EGFP mice was observed in whole mount DRG preparations which are most analogous to the preparation used for electrophysiological recording. Examples shown in Figure 1 are from fixed whole mount preparations after staining for tyrosine hydroxylase (TH). As previously observed in the rat using this method [14], sympathetic fibers could be observed on the surface of the DRG in normal mice, but were not in close association with sensory neurons. As previously reported in both rat and mouse there are small TH-positive neurons in the normal DRG (which are thought to be dopaminergic) and their numbers decrease after the peripheral axotomy [14-22]. Sprouting of TH-positive fibers was observed to start early after spinal nerve ligation, and was quite extensive by postoperative day (POD) 3.

**Figure 2:**
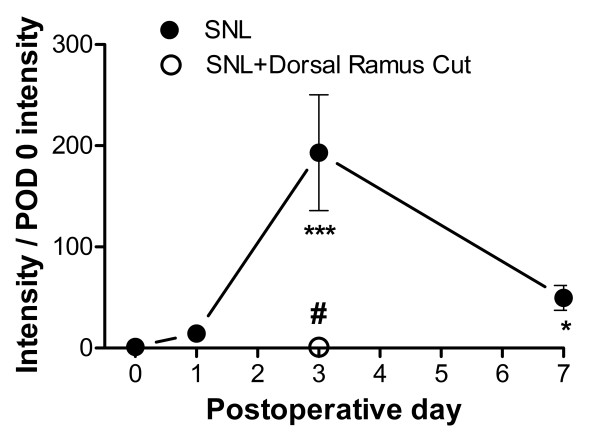
**Time course of sympathetic sprouting**. Average intensity of TH staining from cellular regions of whole mount DRG preparations as shown in Figure 1 is plotted as a function of POD. Data were normalized to the average value in normal DRG. N = 5 - 6 animals/group. *, significantly different from normal (POD 0), Kruskal-Wallis One Way Analysis of Variance on Ranks with Dunn's post test. #, significant difference between POD 3 values with and without cutting of the dorsal ramus in addition to the ventral ramus, Mann-Whitney Rank Sum Test; value with dorsal ramus also cut did not differ significantly from value in normal mice.

As previously reported in rat [14], sympathetic sprouting onto the DRG appeared to come largely from the intact dorsal ramus. This branch of the spinal nerve, which is very small at the lumbar level, separates from the rest of the spinal nerve just after its exit from the intervertebral foramen and innervates posterior structures [23]. In our spinal nerve ligation procedure the dorsal ramus was left intact and only the ventral ramus was cut, as is commonly done in this model. We found that also cutting the dorsal ramus at the time of spinal nerve ligation greatly reduced the amount of sympathetic sprouting observed on POD 3 in mice (Figure 1, 2).

### The incidence of spontaneous activity rapidly increased in axotomized DRG neurons and its profile changed with time after peripheral nerve injury

Microelectrode recordings from intact, acutely excised mouse DRG, were based on the method previously developed for rat DRGs [24,25]. No proteolytic enzymes were used, and the satellite glia were still present. We rarely observed basket formations around small diameter cells, as defined by the diameter observed in the microscope, the capacitance, and the presence of a broad action potential with an inflection on the falling phase. The electrophysiological data presented in this study are all from medium and large diameter neurons.

**Figure 3:**
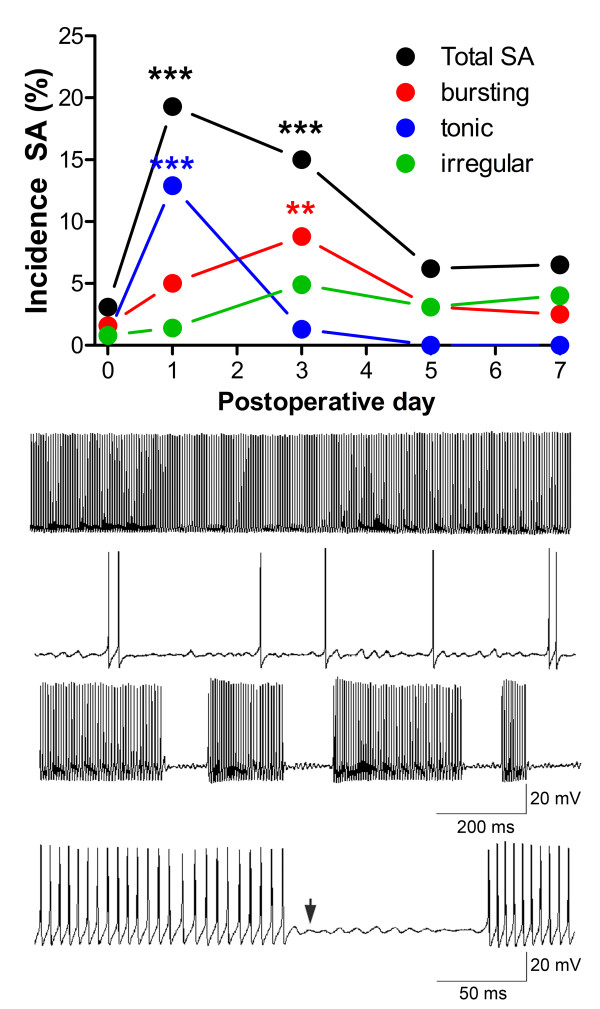
**Overall incidence of spontaneous activity ("Total SA") and of each subtype vs. time after spinal nerve ligation**. *, significantly different from control (POD 0), Fishers exact test. N = 149 to 255 cells per group, with a minimum of 3 different animals per group, for this and Figures 4 and 5. Bottom: examples of tonic (top), irregular (second) and bursting (third) activity. The bursting activity has been expanded to show the subthreshold oscillations observed between bursts (bottom trace).

We first confirmed that, as in rat DRG neurons, SNL caused an increase in the incidence of spontaneous activity in large and medium diameter cells. For these experiments, the green fluorescence near each neuron was not examined; however, the transgenic mice were used in all experiments. In control, uninjured mouse DRG, the incidence of spontaneous activity was very low (3.1%). The overall incidence of spontaneous activity increased sharply after SNL, with a value of 19.3% on POD1, and then declined slowly. As in rat DRG neurons, the pattern of spontaneous activity could be classified as bursting, irregular, or tonic. The form of spontaneous activity changed with time after SNL. Tonic firing prevailed on the first day, but by day 3 bursting was the form most commonly seen (Figure 3). Bursting cells had subthreshold oscillations between bursts (Figure 3).

**Figure 4:**
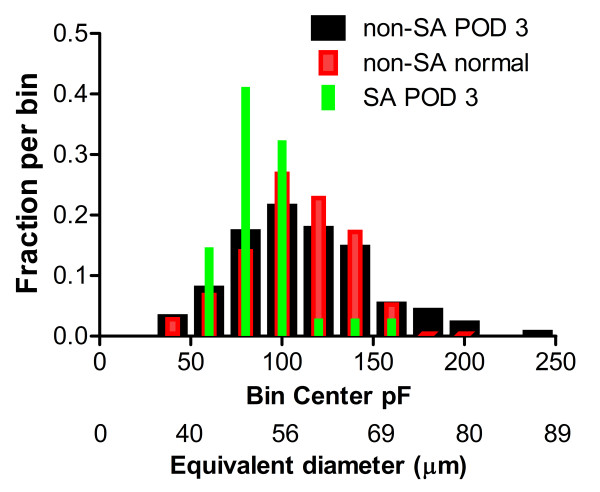
**Distribution of membrane capacitance in spontaneously active vs. not spontaneously active cells (from experiments without green fiber visualization)**. There were not enough SA cells from normal DRG (n = 4) to include in the analysis. Equivalent diameters are based on a sphere with membrane capacitance of 1 μF/cm^2^

Spontaneous activity was primarily observed in cells with a relatively narrower range of capacitance values, those with intermediate capacitance values close to the boundary usually used to distinguish medium and large diameter cells (Figure 4). The very largest cells very rarely showed spontaneous activity. When fit with a Gaussian distribution, spontaneously active cells on POD 3 had a significantly different distribution of capacitance values than cells from the same DRG that were not spontaneously active (P < 0.0001), with a best Gaussian fit having a smaller central value (86 pF compared to 106 pF in non-SA cells) and smaller width (SD = 16.7 pF vs. 36 pF).

Spontaneously active cells tended to have action potentials with no inflection on the falling phase in cells from normal uninjured DRG and from DRG isolated on POD1 thru 7 - overall, only 2% of spontaneously active cells had inflected action potentials. For comparison, the percentage of all cells (spontaneously active or not, in normal cells and on all POD) with inflected action potentials was 29%, a value that did not vary significantly between different POD groups.

When the dorsal ramus was cut along with the ventral ramus, which as shown above greatly decreased sympathetic sprouting as observed on POD 3, the incidence of spontaneous activity on POD 3 was only 1.9%. This was significantly lower (p = 0.0002, Fisher's exact test) than the value observed in the usual SNL procedure in which only the ventral ramus was cut, which was 15% (as shown in Figure 3). In fact, this level of spontaneous activity was not significantly different (p = 1.0, Fisher's exact test) from the level seen in uninjured cells (which was 3.1%, as shown in Figure 3). The 2 of 104 cells that showed spontaneous activity when the dorsal ramus was also cut were both of the bursting type.

### The excitability of DRG neurons was increased after spinal nerve injury

**Figure 5:**
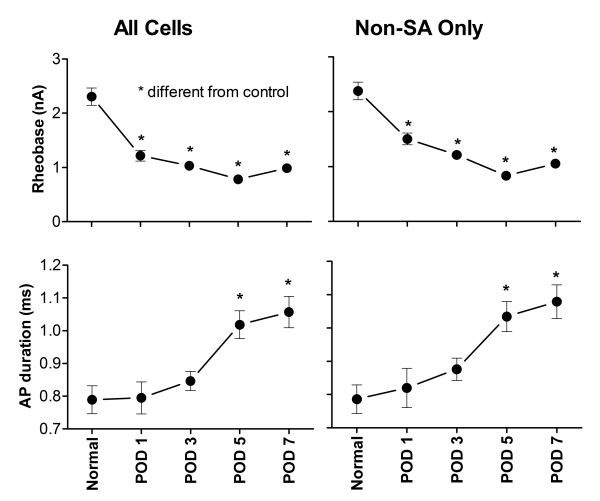
**Two measures of excitability are increased in large and medium diameter mouse DRG neurons after spinal nerve ligation**. *, significantly different from normal, uninjured mice. (Kruskal-Wallis One Way ANOVA on Ranks with Dunn's post test). Data is from recordings in which all cells were systematically recorded, without examining fluorescence. For spontaneously active cells, rheobase was defined as 0. Spontaneously active cells were omitted from the analysis to create the graphs on the right. Error bars (SEM) if not visible are smaller than the symbols.

We also confirmed that, as in rats [26], spinal nerve ligation increased the excitability of mouse DRG neurons in the axotomized DRG. As shown in Figure 5, mouse DRG neurons displayed reduced rheobase as early as POD 1. Action potentials became broader, but this effect was not observed until POD 5.

The marked decrease in rheobase observed after SNL was not simply due to the presence of more spontaneously active cells (in which the rheobase was defined as zero). In comparing only cells without spontaneous activity, rheobase was still significantly lower after SNL, on all days examined (Figure 5, right).

Although cutting the dorsal ramus in addition to the ventral ramus greatly reduced the incidence of spontaneous activity, the lower rheobase observed in these cells, 1.15 ± 0.11 nA (N = 102 cells without spontaneous activity), was not significantly different from the rheobase observed in POD 3 cells from animals in which only the ventral ramus was cut (1.2 ± 0.08 nA, N = 192 cells without spontaneous activity, Figure 5, p = 0.29). The comparison was also not significant when all cells were included instead of just cells lacking spontaneous activity. These rheobase values were significantly lower than those observed in cells from normal uninjured DRG.

### The DRG neurons surrounded by sympathetic nerve baskets or rings were more likely to be spontaneously active and bursting than other neurons

**Figure 6:**
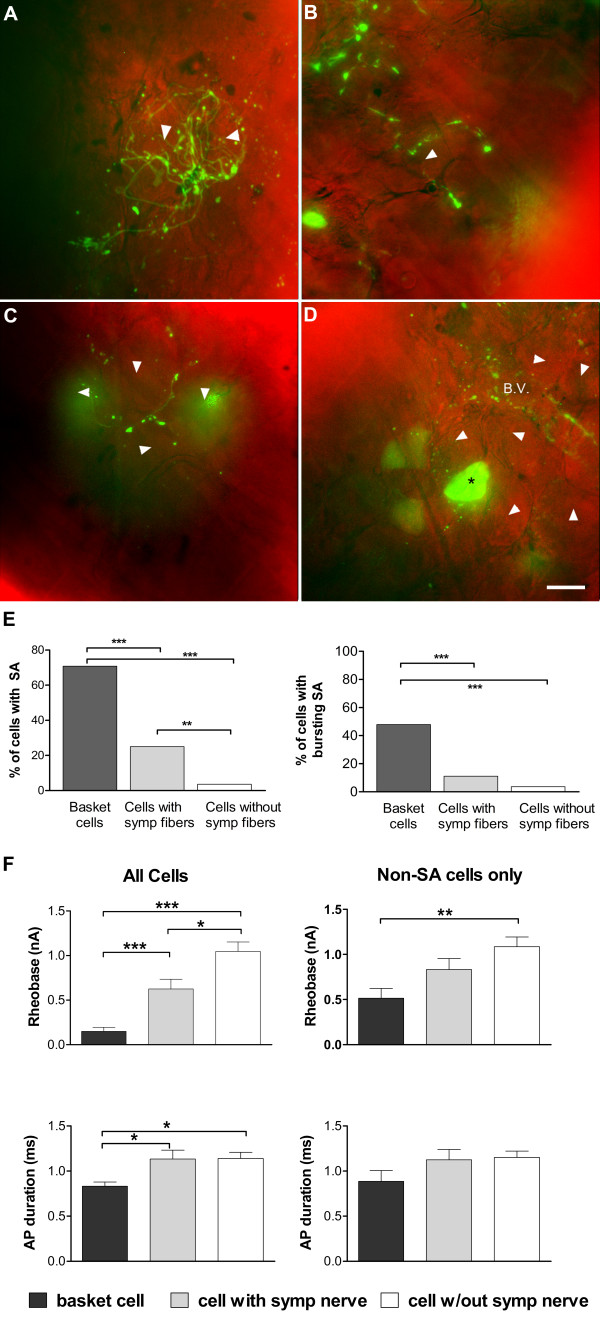
**Electrophysiological properties of neurons whose surrounding sympathetic fibers were examined before recording**. A - D: arrowheads indicate examples of cells classified has having sympathetic baskets (A) or rings (B) (such cells were combined into a single group of "basket cells"); cells with nearby but less elaborated sympathetic fibers (C); and no nearby sympathetic fibers (D), based on observation of EGFP fluorescence during the recording session. B.V. in (D) indicates a blood vessel. Some small TH-positive neurons are also seen (see text). EGFP fluorescence is shown in green and is from combined layers of a confocal image. Neurons are not counterstained because the images are from live preps used for recording; however, a bright field images of the cells taken in a single plane is shown in the red channel. (E) Basket cells have much higher incidence of spontaneous activity, primarily of the bursting pattern. Overall incidence of spontaneous activity (left), as well as incidence of bursting (right), were significantly higher in basket cells compared to either cells with nearby sympathetic fibers but not making a basket/ring formation, or to cells without any nearby sympathetic fibers (Fisher's exact test). (F) Cells (left) with sympathetic basket formations have lower rheobase (F, top) and shorter AP duration (F, bottom) compared to cells with nearby fibers (not forming a basket or ring) or no nearby fibers; the difference in rheobase is still seen when only non-spontaneously active cells are considered. (ANOVA on ranks with Dunn's post test).

POD 3, when sympathetic sprouting was well developed and spontaneous activity was still elevated, was chosen for subsequent studies of individual neurons defined during the recording as being surrounded by sympathetic basket (Figure 6A) or ring formations (Figure 6B), (n = 34), having nearby sympathetic fibers but not a basket formation (Figure 6C; n = 16), or having no nearby sympathetic sprouts (Figure 6D; n = 63) as described in Methods.

As shown in Figure 6, cells surrounded by basket or ring formations were highly likely to be spontaneously active, compared to either the whole population examined without examination of GFP fluorescence (data presented in Figure 3) or to cells without basket formations from DRG in which EGFP fluorescence was observed during recording (Figure 6). The majority of this spontaneous activity was of the bursting pattern. The incidence of all types of spontaneous activity in basket cells was 71%, and the incidence of bursting was 48%. This compares to values of 15% incidence of spontaneous activity and 9% incidence of bursting on POD3 in the total population of DRG cells without examining GFP fluorescence (Figure 3).

The rheobase and action potential width also differed between basket cells and neurons with only sympathetic fibers or no nearby sympathetic fibers (Figure 6, bottom).

The significantly lower rheobase observed in basket cells was not entirely due to the inclusion of a large number of spontaneously active cells for which the rheobase was defined as zero. As shown in Figure 6 (right), differences in rheobase were significantly different between basket cells and cells lacking nearby sympathetic fibers even when considering only the subset of cells without spontaneous activity. However, the shorter action potential duration observed in basket cells may have been due to the high percentage of spontaneously active basket cells. The differences disappeared when only non-spontaneously active cells were considered (Figure 6, right), and, as in the data from unlabeled cells, spontaneously active cells showed noninflected action potentials regardless of the presence or absence of nearby sympathetic fibers (overall 2.4% of spontaneously active cells, vs. 36% of non-spontaneously active cells, had inflected action potentials in this set of experiments with labeled cells).

**Figure 7:**
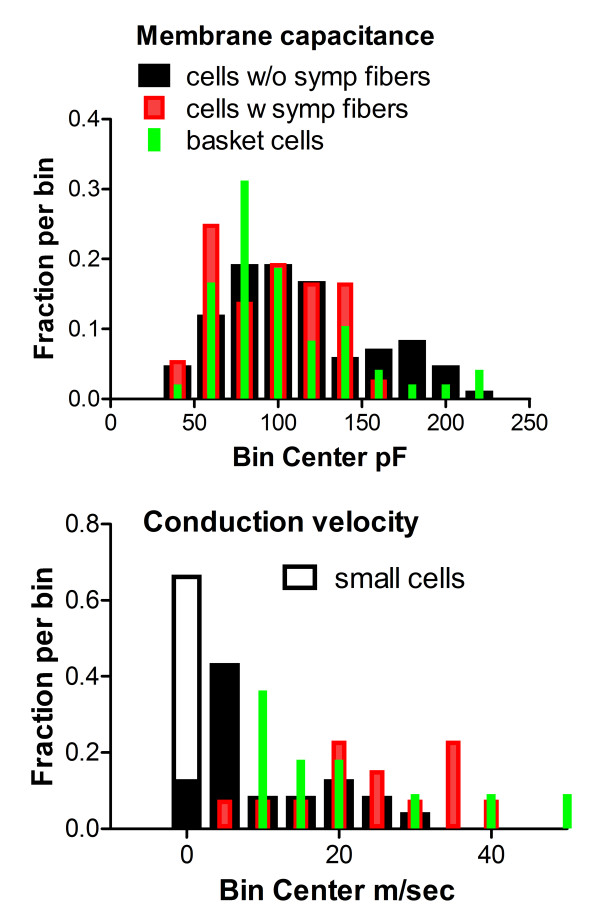
**Characteristics of basket cells**. Top: membrane capacitance distribution for all cells studied after identification of sympathetic fiber status. Bottom: distribution of dorsal root conduction velocities measured in a subset of the same cells. No basket cells had conduction velocities below 9.5 m/sec. For comparison, conduction velocities measured in a subset of cells, defined by our criteria as small cells and excluded from the other analyses, are also shown in the conduction velocity histogram (open bars).

Neurons with basket formations had a capacitance distribution which was relatively underrepresented in the largest cells, compared to the cells with only nearby fibers or with no nearby fibers (Figure 7). In a subset of cells the conduction velocity in the dorsal root was measured. Although the higher values of conduction velocity cannot be considered to be very accurate measurements, due to the shortness of the dorsal root in these preparations, it was clear that all the basket cells fell into a range consistent with having myelinated axons. No basket cells had conduction velocities lower than 9.5 m/s. In contrast slower conduction velocities could be readily measured in some of the cells with nearby sympathetic fibers or no nearby fibers. For comparison, conduction velocities measured in the small cells (that have been otherwise excluded from the overall analysis of electrophysiological experiments presented here), are included in the conduction velocity histogram shown in Figure 7.

### Neurons surrounded by sympathetic nerve baskets or rings were more likely to express nociceptor markers

**Figure 8:**
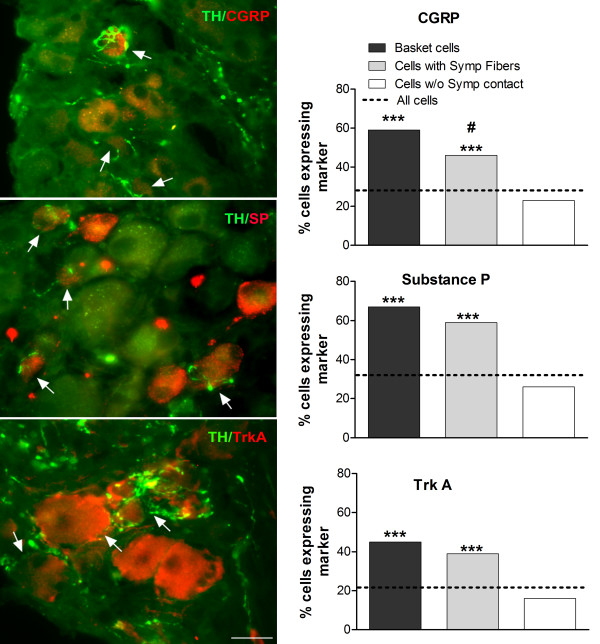
**Distribution of nociceptive markers in cells with and without sympathetic contacts**. DRG sections were stained for the indicated marker (red) and for TH (green) on POD3. Left: examples of dual staining from fixed DRG sections. Arrows indicate examples of cells expressing the indicated marker along with sympathetic basket formations or nearby fibers. (Scale bar = 25 μm). Right: summary of percentage of cells in each class expressing the indicated marker. ***, percentage of cells with basket/ring formations or with nearby sympathetic fibers expressing each marker was significantly higher (p < 0.001) than the percentage observed in cells lacking sympathetic contacts. #, significant difference (p < 0.05) between the basket cells and cells with nearby fibers (Chi-square test). The dotted lines indicating the overall percentage of cells expressing each marker on POD 3 are close to the values for cells with no sympathetic fibers because only 18 - 22% of cells have any sort of sympathetic contact. Data are combined from 3 separate animals for each marker; findings were similar within each individual group. N values for each group are given in Table 1.

In order to further characterize the cells that received sympathetic inputs, dual staining for TH and one nociceptor marker (CGRP, substance P, or TrkA receptor) was carried out in sectioned DRG tissue in normal DRG and on POD 3. As shown in Figure 8, cells with surrounding sympathetic fibers or basket formations were more likely to express one of these markers (also see Table 1). Conversely, cells expressing one of these markers were more likely to also have surrounding sympathetic fibers or basket formations than were cells without any sympathetic fibers. For substance P and TrkA, there was no significant difference between cells with sympathetic baskets or cells with nearby fibers in the fraction of cells expressing the marker though the tendency was for basket cells to have a higher fraction expressing the marker than cells with nearby fibers (p = 0.07 and 0.22 respectively). For CGRP the percentage of basket cells expressing the marker was significantly higher than the percentage of cells with nearby fibers expressing the marker (p = 0.02), however, both groups were significantly more likely to express CGRP than were cells with no nearby sympathetic fibers. The numbers of cells in each individual group are presented in Table 1. The size distribution of cells expressing each marker is shown in Additional file 1, Figure S1. As in previous studies (see discussion), substance P was primarily observed in small cells while CGRP and TrkA had broader distributions. On POD 3, the percentage of cells expressing substance P was significantly higher than in normal DRG; the percentages of cells expressing CGRP or TrkA were significantly lower (Table 1). The data did not definitively show a selective upregulation of these markers in larger cells.

## Discussion

Because we could visualize sympathetic fibers in live tissue, using the transgenic mouse allowed us to efficiently identify and record from basket cells which are relatively rare. The present results validate the use of pTH-EGFP mice to investigate sympathetic-sensory coupling under pathological conditions. The rapid appearance of sprouting sympathetic fibers (by POD 3) after spinal nerve ligation that we observed in our whole mount preparations is consistent with results obtained with higher resolution, thin section methods in rats [27]. The rapid increase of spontaneous activity (especially, bursting activity) in large diameter cells with high conduction velocities, and their relatively narrow size distribution, is also similar to that previously reported in rats [14,26,28,29]. It is worth noting that cells with high conduction velocities but relatively small diameters have been described as belonging to the small group of nociceptive Aβ-fiber neurons in guinea pig neurons [30]. Cells with similar properties were also observed to express Substance P only after chronic constriction injury in mice [31] though the N values were too small to be significant. The great reduction in sprouting at POD3 if the dorsal ramus is cut along with the ventral ramus is also similar to observations made in rat [14]. It is of interest that cutting the grey ramus to the L5 DRG, which also reduces sprouting within the DRG, can greatly reduce SNL-induced pain behaviors in the rat [14,32]. Co-localization of sympathetic sprouts with spontaneously active neurons was demonstrated using the rat sciatic nerve transection model to induce sprouting [33]. These comparisons suggest that studies of sympathetic sprouting give similar results in mice and rats, enhancing the usefulness of transgenic mice in studying sympathetic sprouting.

Sympathetic basket formations around sensory neurons after peripheral nerve injury or inflammation, as first observed by McLachlan et al.[1], initially generated much interest because they provided a possible anatomical basis for sympathetically enhanced or maintained pain states. However, their relevance to pain models has been questioned in part because of conflicting behavioral studies (see discussion in ref [14]), and in part because basket formations are relatively rare. The data presented in this study provide evidence for important functional roles for these sympathetic sprouts. We find that at a time early after injury (POD 3) sensory neurons with basket formations are much more excitable than neighboring neurons without basket formations; and, in particular, the basket cells have a very high incidence of spontaneous activity (71% showed spontaneous activity, primarily of the bursting pattern). In the data from DRG sections stained for TH, the average incidence of basket formations was 6.8% (Table 1). For comparison, the incidence of spontaneous activity in all DRG cells at this time point was 10% (Figure 3, after allowing for the fact that small cells were excluded from the electrophysiological data). This suggests that over half of the overall spontaneous activity may be attributed to basket cells, despite their overall rarity. In turn, spontaneous activity has been shown to play key roles in pain models, including roles in satellite glia activation, neurotrophin release, spinal cord sensitization, and establishment of pain behaviors[28,34-37]. Additional evidence for functional relevance of sympathetic sprouting comes from our observations that cells that have nearby sympathetic fibers, but not a full-blown basket or ring formation, have an intermediate electrophysiological phenotype between that of basket cells and cells lacking any nearby sympathetic fibers. Though studies based on microscopy have tended to focus on and quantitate only the basket formations, the cells with the lower density nearby fibers are more numerous (11.5% vs. 6.8%; Table 1) and insofar as the presence of these nearby fibers is also associated with a more excitable phenotype, it seems that these structures may also contribute to a functional role for sympathetic sprouting.

The data presented here show an association between spontaneous activity in vitro and the presence of sympathetic fibers around the neuron. This suggests a highly localized interaction between the sympathetic fibers and nearby sensory neurons - neighboring cells lacking nearby fibers have different properties. This may reflect long-lasting, pro-excitatory interactions in vivo that are preserved in our acutely isolated in vitro preparation, and/or may indicate ongoing release of excitatory sympathetic transmitters in our in vitro preparation. Some evidence for the latter possibility was obtained in rat DRG, in medium but not large diameter cells [14]. Do spontaneously active neurons attract sympathetic fibers, or do nearby sympathetic fibers enhance excitability and help drive spontaneous activity? Or could both processes occur in a positive-feedback cycle? This study provides evidence for interactions going in both directions. On the one hand, spontaneous activity is highest on day 1, before much sympathetic sprouting has occurred, suggesting spontaneous activity cannot be initiated by sympathetic inputs. However, we also found that simply cutting the small dorsal ramus in addition to the larger ventral ramus that is usually ligated in the SNL model, reduced sprouting on POD3 and also dramatically reduced spontaneous activity to levels seen in control animals. Insofar as spontaneous activity is initiated by axonal ligation, cutting both dorsal and ventral ramus would have been expected to increase spontaneous activity, not decrease it, though such an effect would have been quite small given the very small number of lumbar DRG neurons with projections in the dorsal ramus (less than 3% in rat [14]). This suggests that it is more likely the large reduction in sympathetic sprouting that explains the observation that cutting the dorsal ramus greatly reduces spontaneous activity.

The literature describes a number of possible mechanisms for the postulated two-way interaction between spontaneous activity and sympathetic sprouting. Spontaneously active cells may release neurotrophic factors that attract sympathetic fibers, either directly or via activation of surrounding satellite glia cells that then synthesize neurotrophic factors (reviewed in [36]). In the rat sciatic nerve transection model, blocking nerve activity early after injury reduces sympathetic sprouting, and increasing activity increases sprouting[33]. Conversely, sensory neurons (and their associated satellite glia) also express receptors for sympathetic neurotransmitters norepinephrine and ATP that have excitatory effects on neurons, particularly after injury or inflammation [38-40], and sympathectomy reduces SNL-induced spontaneous activity recorded in rat dorsal root fibers [41].

The membrane capacitance distributions suggested that spontaneously active cells as well as basket cells were in the size range near the boundary between medium and large diameter cells. The very largest cells were not generally spontaneously active and did not have basket formations. However, in experiments in which conduction velocity was measured, basket cells always had conduction velocities above 9 m/s; though the higher velocities were not accurately measured due to the shortness of the stimulated dorsal root and the physiological temperature used. These velocities were clearly distinct from the much slower, accurately measured values seen in a subset of cells including small cells. The conduction velocities and size distribution suggested that spontaneously active cells and cells with basket formations might overlap with myelinated nociceptors. Though most nociceptors are small cells with nonmyelinated axons, a significant minority of cells conducting in the Aαβ range show nociceptive (high) thresholds and project into lamina I/II of the spinal cord [42,43]. In rat it is estimated that 20% of Aαβ cells are myelinated nociceptors [42]. Consistent with this possibility, we observed that several commonly used nociceptive markers - TrkA, CGRP, and substance P - were markedly enriched in basket cells compared to cells lacking nearby sympathetic fibers. TrkA in particular has been proposed as a marker for the myelinated nociceptors [42], and an association of TrkA with sprouting induced by NGF overexpression in mice has been reported [44]. However, there are also numerous mechanisms by which Aαβ neurons that are not normally nociceptive can contribute to abnormal pain states [45]. Further studies are needed to more precisely define the physiological roles and projections of the basket cells and spontaneously active cells.

We found that substance P was largely confined to small and medium diameter cells (Additional file1, Figure S1), while CGRP and TrkA had a somewhat broader distribution, similar to previous studies in both rats [46-49] and mice [50,51]. Some studies, primarily in rat, have shown upregulation of substance P and/or CGRP specifically in medium or large diameter neurons in various pain models including SNL [50-54]. Our immunohistochemical data did not provide clear evidence for this phenomenon, possibly because we studied a much earlier time point than most of the above studies or because the time course may differ in mice. Comparison of cells with upregulated substance P or CGRP in previous studies, with the basket cells described in this study, is confounded by the fact that the basket cells described here (especially the spontaneously active ones) would have been defined as having Aβ fibers in functional studies but probably would be classified as "medium" diameter cells in many microscopy studies; the categorization of cells as small, medium, or large diameter does not correlate very precisely with C, Aδ, and Aβ conduction velocities respectively [31]. The present study was not designed to specifically address the question of selective upregulation of substance P or CGRP in medium and large diameter cells, but does show that these nociceptive markers are enriched in cells with nearby sprouting sympathetic fibers.

## Conclusions

When sympathetic sprouting into the dorsal root ganglion is induced by spinal nerve ligation, sensory neurons surrounded by sympathetic fibers are much more likely to exhibit spontaneous activity and increased excitability. These cells have myelinated axons but have sizes near the boundary between medium and large cells, and are enriched in nociceptive markers. This suggests that sympathetic sprouts have localized, functional, excitatory interactions with sensory neurons which may contribute to pathological pain.

## Methods

### Animals

Transgenic mice expressing EGFP under the TH promoter [55,56] were obtained from the Mutant Mouse Regional Resource Centers and bred via hemizygous x wildtype (ND4 Swiss Webster) crossings in the local animal facility. Genotyping was performed using DNA isolated from tail clips. Mice were housed four per cage under a controlled diurnal cycle of 12 h light and 12 h dark with free access to water and food. The ambient environment was maintained at constant temperature (22 ± 0.5°C) and relative humidity (60-70%). All the surgical procedures and the experimental protocol were approved by the Institutional Animal Care and Use Committee of the University of Cincinnati (Cincinnati, OH).

### Spinal nerve ligation

Female transgenic mice (pTH-EGFP) weighing 20-30 g at the time of surgery were anesthetized with isoflurane. An incision was made on the back between L2 and S1. The L4 and L5 spinal nerves were exposed and tightly ligated with 6-0 silk and cut about 2 mm distal to the ligature. The procedure was based on the original version of this pain model in rat [57] in which the L5 and L6 spinal nerves were ligated; except that as many subsequent researchers have done, the spinal nerves were also cut after being ligated; in addition, cutting L4 and L5 spinal nerves in the mouse has been proposed on an anatomical basis to most closely match cutting the L5 and L6 spinal nerves in the rat [58].

### Recording from whole excised DRG preparation

Intracellular recording was performed on sensory neurons with or without surrounding sympathetic fibers in whole DRG preparations isolated from normal or SNL mice at POD 1, 3, 5 or 7 as indicated. The ipsilateral L5 or L4 DRG was dissected out of the mouse under barbiturate anesthesia, placed in the recording chamber and mounted on the stage of an up-right microscope (BX70-WI, Olympus). A U-shaped stainless steel rod with 3 pieces of fine nylon filaments crossing from one side to the other was used to gently hold the ganglion in place within the recording chamber. Reducing the size of this holder was the primary change required to adapt the recording method used in rat DRG [14,24] to the smaller mouse DRG. The DRG was continuously perfused with artificial cerebrospinal fluid (ACSF) bubbled with 95%O_2 _5%CO_2 _containing (in mM): NaCl 130, KCl 3.5, NaH_2_PO_4 _1.25, NaHCO_3 _24, Dextrose 10, MgCl_2 _1.2, CaCl_2 _1.2 (pH = 7.3). The temperature was maintained at 36 ± 1°C. Intracellular, electrophysiological recordings were made with microelectrodes filled with 3 M KCl,10 mM HEPES, 10 mM EGTA (pH = 7.2) coated with Sigmacote (Sigma, St. Louis, USA). Satisfactory recordings were obtained with electrodes of 30-50 MW from large- (> 40 mm) and medium-sized (< 40 mm) neurons, classified by the diameter of the soma. Small diameter neurons as defined by capacitance and the presence of a broad inflected action potential, approximately 33% of the cells that could be impaled from the DRG surface in our preparation, were observed to rarely receive sympathetic sprouts under the conditions used and rarely manifested spontaneous activity. Hence data obtained in recordings from these cells are not presented in this study, which focuses on medium and large diameter cells. The electrophysiological data were collected with the use of single-electrode continuous current-clamp (Axoclamp 2B, Molecular Devices, Sunnyvale, CA, USA) and analyzed with pClamp 9 software (Molecular Devices). In experiments to determine the incidence of spontaneous activity, individual DRG neurons were first impaled with a recording electrode. If spontaneous activity was absent during the first 60 sec of the impaling, incremental currents (up to 5 nA) were then injected to ensure that action potentials could be evoked indicating a healthy cell. If any spontaneous activity was present, then we waited for 3 min to ensure that the activity was not caused by penetrating the somata with the sharp electrode. Next, the following parameters were measured using a series of current pulse injections: the threshold current (rheobase), action potential (AP) threshold and duration (measured at threshold), resting membrane potential (Vm) and input resistance (Rin, measured from the size of the voltage response to hyperpolarizing current injections), and capacitance (measured by fitting a single exponential function to the early part of the response to hyperpolarizing current injection) of the recorded DRG cell. In some experiments, the dorsal root (8 - 13 mm) was pulled into a suction electrode for applying single extracellular current stimuli to evoke action potentials for measurement of conduction velocities. All electrophysiological data presented are from transgenic mice, though the green fluorescence was not observed in every experiment.

For experiments in which cells were identified before recording as having basket formations or nearby sympathetic fibers, green fluorescent protein was imaged using a spinning disk confocal, so as to observe the live tissue with relatively low light intensity to reduce any possible tissue damage caused by the laser. The surface of the DRG was observed for fluorescence before recording. Any neuron identified as being surrounded by sympathetic basket or having nearby sympathetic fibers was localized for recording as quickly as possible (usually within 1 min). The light source was turned off during recording and turned on again to take a picture of the cell just after recording to document the status of sympathetic fiber sprouting. In addition to recording neurons with nearby sympathetic fibers, some neurons not having sympathetic fiber baskets or nearby fibers were randomly picked for recording after examining fluorescence, to eliminate the possibility that fluorescent light exposure per se might evoke spontaneous activity in DRG neurons. Because in these experiments cells were chosen based on their sympathetic fiber status, the relative numbers of cells in each group will not reflect the overall incidence of each cell type.

### Immunostaining of TH-positive sympathetic nerve fibers in whole-mount DRG

Mice were anesthetized with pentobarbital sodium and fixed by perfusing with 20-30 ml of Zamboni's fixative through the left ventricle of the heart. Whole DRGs were first incubated with rabbit anti-TH (1:1000) (Pel-Freeze, Rogers, AR) 48 h at 4°C followed by a reaction with Alexa Fluor 594 conjugated goat anti-rabbit secondary antibodies (1:1000) (Invitrogen, Carlsbad, CA). Using SlideBook 4 Digital Microscopy Software (Intelligent Imaging Innovation, Denver, CO), confocal images were captured and stored in a computer for later analysis. The microscope was the same as used for recording, and the confocal was used to reduce background fluorescence. For quantification, the summed intensity of TH signal in the cellular regions of the whole mount DRG was measured via using Slidebook 4.1 imaging acquisition software (Intelligent Imaging Innovation, Denver, CO).

### Immunostaining of TH-positive sympathetic nerve fibers and nociceptor markers in sectioned DRG

On postoperative day 3 or in normal mice as indicated, mice were anesthetized with pentobarbital sodium (40 mg/kg, i.p.) and were fixed by perfusing 50-100 ml of Zamboni's fixative (4% paraformaldehyde in 0.1 M phosphate buffer, pH = 7.4) through the left ventricle of the heart.

Ipsilateral DRGs (L4 and L5) were removed. Tissue was post-fixed in the perfusion fixative for 2 hours at room temperature. The ganglia were horizontally sectioned with a Cryostat at thicknesses of 10 μm. DRG sections were incubated in sheep anti-TH (Abcam, Cambridge, MA) at a dilution of 1:500 and rabbit anti TrkA, or CGRP, or SP all at a dilution of 1:500 (Abcam) overnight at 4°C, followed by reaction with secondary antibodies, donkey anti rabbit conjugated to Alexa Fluor 594 and donkey anti sheep conjugated to Alex Fluor 488 (1:1000, Invitrogen, Carlsbad, CA) for 1 hour at room temperature. After drying, the sections were mounted on coverslips with Vector Hard Set mounting medium (Vector Laboratories Inc., Burlingame, CA, USA).

Slides from control and experimental groups were labeled with numbers so that the person performing the image analysis was blinded as to the experimental group. In addition, all images were captured and analyzed by an investigator other than the one who performed immunohistostaining to avoid possible bias. Images from ~10 sections of each DRG were captured under a confocal microscope using Slidebook 4.1 imaging acquisition software (Intelligent Imaging Innovation, Denver, CO). The number of neurons surrounded or innervated by sympathetic fibers was counted and then normalized by the total number of neurons in the analyzed image area to give a percentage of neurons with basket formations or nearby sympathetic fibers. In double staining experiments, neurons expressing TrkA, CGRP or SP were counted and classified as having basket formations, nearby sympathetic fibers, or no nearby fibers.

### Data analysis

Data are expressed as the mean ± the standard error of the mean. ANOVA or Kruskal-Wallis One Way ANOVA on ranks, was used compare multiple groups; t-test or Mann-Whitney sum test was used to compare two groups; the nonparametric test was chosen whenever the data were not normally distributed. The test used in each case is indicated in the text or figure legend. Fisher's exact test was used to test the significance of differences in 2 × 2 tables except for tables with larger N values for which the Chi Square test was used. For electrophysiological data, all experimental groups included cells from at least 3 different animals. Quantification of immunohistochemical data was also done using sections or samples from at least 3 different animals per experimental group. Significance was ascribed for p < 0.05. Level of significance is indicated by the number of symbols, e.g., *, p = 0.01 to < 0.05; **, p = 0.001 to 0.01; ***, p < 0.001.

## List of Abbreviations

AP: action potential; CGRP: Calcitonin gene related peptide; DRG: dorsal root ganglion; EGFP: enhanced green fluorescent protein; POD: post-operative day; SNL: spinal nerve ligation; SP: Substance P; TH: tyrosine hydroxylase.

## Competing interests

The authors declare that they have no competing interests.

## Authors' contributions

WX conducted electrophysiological and microscopy experiments, data analysis, experimental design, and manuscript revision. JAS contributed to experimental design, data analysis, and drafting of the manuscript. JM contributed to immunohistochemistry experiments and maintained the transgenic mouse line. J-MZ contributed to experimental design, manuscript revision, and data analysis. All authors read and approved the final manuscript.

## Supplementary Material

Additional file 1 **Size distribution of cells expressing nociceptive markers in normal and SNL DRGs**. Size distribution histograms from normal DRG (left) and DRG 3 days after SNL (right) for cells labeled for substance P (top), CGRP (middle) and TrkA (bottom). The overall distribution for all cells is shown in black; the distribution for cells expressing the marker is shown in red (normal DRG) or green (SNL DRG). The latter distributions were scaled to reflect the overall percentage of cells expressing the marker (see Table 1). Click here for file
