# Supplementary figures and images for: Highly localized interactions between sensory neurons and sprouting sympathetic fibers observed in a transgenic tyrosine hydroxylase reporter mouse
Source: Mol Pain. 2011 Jul 27;7:53. doi: 10.1186/1744-8069-7-53 (PMC3152901; doi:10.1186/1744-8069-7-53)

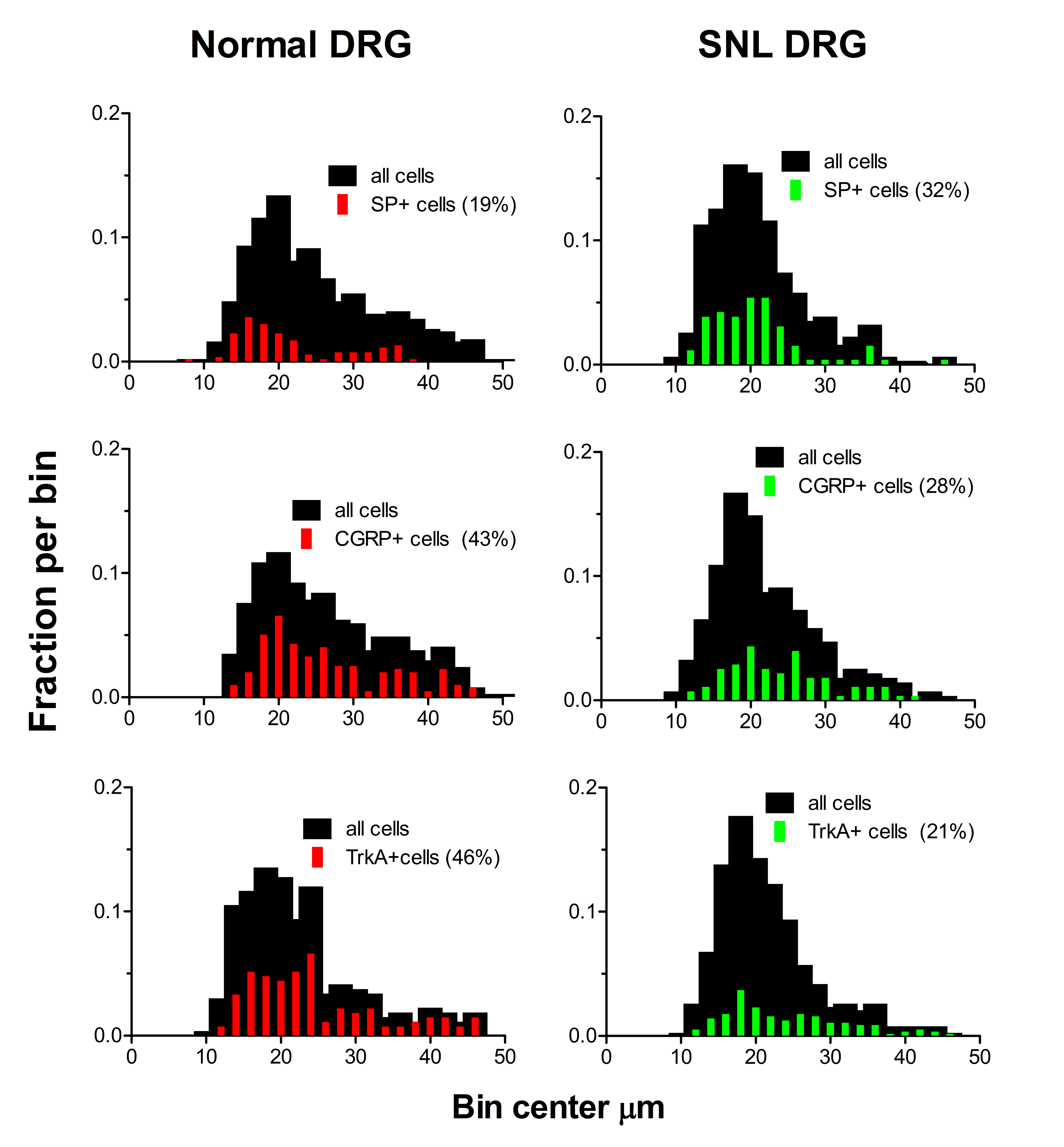

Supplement: Additional file 1 — Size distribution of cells expressing nociceptive markers in normal and SNL DRGs. Size distribution histograms from normal DRG (left) and DRG 3 days after SNL (right) for cells labeled for substance P (top), CGRP (middle) and TrkA (bottom). The overall distribution for all cells is shown in black; the distribution for cells expressing the marker is shown in red (normal DRG) or green (SNL DRG). The latter distributions were scaled to reflect the overall percentage of cells expressing the marker (see Table 1). [file 1744-8069-7-53-S1.TIFF]
